# Transient Mitomycin C-treatment of human corneal epithelial cells and fibroblasts alters cell migration, cytokine secretion, and matrix accumulation

**DOI:** 10.1038/s41598-019-50307-9

**Published:** 2019-09-25

**Authors:** Sonali Pal-Ghosh, Gauri Tadvalkar, Verna Rose Lieberman, Xiaoqing Guo, James D. Zieske, Audrey Hutcheon, Mary Ann Stepp

**Affiliations:** 1 George Washington University School of Medicine and Health Sciences, Department of Anatomy and Cell Biology, 2300 I St. NW, Washington, DC 20037 USA; 2 Schepens Eye Research Institute/Massachusetts Eye and Ear, Department of Ophthalmology, Harvard Medical School, 20 Staniford St, Boston, MA 02114-2500 USA; 3 George Washington University School of Medicine and Health Sciences, Department of Ophthalmology, 2300 I St. NW, Washington, DC 20037 USA

**Keywords:** Extracellular matrix, Cells

## Abstract

A single application of Mitomycin C (MMC) is used clinically in ophthalmology to reduce scarring and enhance wound resolution after surgery. Here we show *in vitro* that a 3-hour MMC treatment of primary and telomerase immortalized human corneal limbal epithelial (HCLE) cells impacts their migration and adhesion. Transient MMC treatment induces HCLE expression of senescence associated secretory factors, cytokine secretion, and deposition of laminin 332 for several days. Transient MMC treatment also reduces migration and deposition of transforming growth factor-β1 (TGFβ1)-stimulated collagen by corneal fibroblasts. Using conditioned media from control and MMC treated cells, we demonstrate that factors secreted by MMC-treated corneal epithelial cells attenuate collagen deposition by HCFs whereas those secreted by MMC-treated HCFs do not. These studies are the first to probe the roles played by corneal epithelial cells in reducing collagen deposition by corneal fibroblasts in response to MMC.

## Introduction

Mitomycin C (MMC) is an anticancer drug discovered in the 1960’s^1^ which is used in oncology to treat various types of solid tumors since it does not induce multidrug resistance^2^. The half-life of MMC within tumors is short, with times cited ranging from 17 minutes to an hour; within 6–8 hours, levels are below detection^3^. After proving that co-culturing epithelial cells with fibroblasts enhanced retention of the epithelial progenitor cell phenotype, methods to prevent fibroblasts from proliferating were developed, including lethal irradiation and the use of MMC^4^. By the 1990’s, MMC was being used to generate “feeder layers” of fibroblasts to support the growth of primary epidermal and corneal epithelial cells^5–7^. Fibroblasts treated with low doses of MMC assume an irreversible phenotype termed drug induced accelerated senescence^8,9^. Senescence induces the secretion of proteases, cytokines and enzymes referred to as the senescence associated secretory phenotype or SASP^10,11^.

In ophthalmology, MMC was initially used topically to treat pterygium and squamous cell carcinoma of the conjunctiva^12^ and in glaucoma filtration surgery^13^. MMC is also used to reduce scarring and enhance functional recovery after refractive surgery^14,15^. Care is taken by refractive surgeons to avoid application of MMC to the corneal and/or limbal epithelium due to concerns with the impact of MMC on epithelial cell proliferation and reepithelialization. Oncologists treating tumors of the conjunctiva and pterygia apply 0.02% MMC topically 4x a day for 14 days followed by a rest interval of 2 weeks before repeating the cycle 3 or more times as needed to achieve tumor regression^16–18^. While corneal stem cell deficiency becomes a concern among ophthalmic oncologists when MMC treatments last 75 days or more^17^, topical MMC treatment remains the standard of care for these conditions. Based on these considerations, a single 3 minute topical application of 0.02% MMC was used at the time of injury in mice to enhance reinnervation of the corneal sensory nerves after wounding^19^ and to study gene expression in the corneal epithelium and stroma after wounding^20^.

The primary clinical tissue targeted by MMC in refractive surgery is the corneal stroma where it reduces fibrosis and enhances clinical outcomes^14,15^. From studies of feeder layers, we know that MMC induces senescence in fibroblasts; after refractive surgery, cells adjacent to the treatment site proliferate, migrate, and close wounds^15^. These stromal cells would be exposed to factors that were secreted by senescent MMC-treated fibroblasts that can persist for weeks *in vivo* and *in vitro*^9,11^. At present it is unknown if ocular surface epithelial cells also respond directly to MMC by assuming a senescent phenotype *in vitro*; however, by treating the mouse ocular surface with a single application of MMC, gene expression changes were induced^21^ hours post treatment within both the epithelium and stroma which were consistent with a senescent phenotype^20^. Whether corneal epithelial cells *in vitro* respond similarly is not known. The impact of proteins, lipids, and other molecules secreted by MMC-treated corneal epithelial cells on corneal stromal fibroblasts and their ability to secrete collagen and other extracellular matrix (ECM) proteins also is not known. Therefore, in the present study we determined the impact of MMC treatment on primary and hTERT immortalized human corneal epithelial cells (HCLE), and the impact of molecules secreted by MMC-treated HCLE cells on collagen deposition by human corneal fibroblasts.

## Results

### Transient MMC treatment (3 hour) reduces HCLE and PHCE cell migration and adhesion

**Figure 1:**
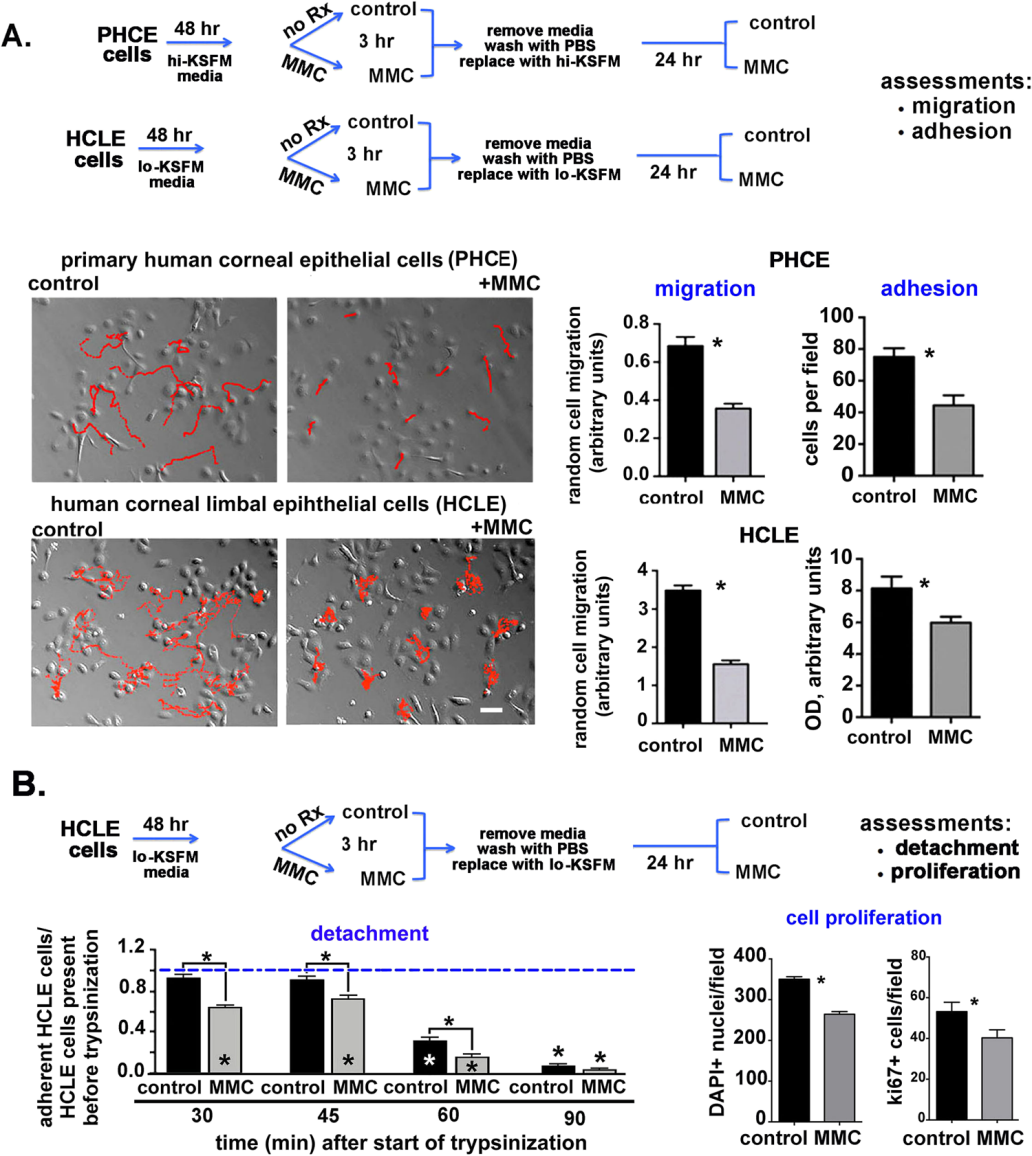
Transient treatment of primary (PHCE) and hTERT immortalized human corneal epithelial cells (HCLE) with MMC for 3 hours reduces migration, adhesion, and proliferation. (**A**) Control and MMC treated PHLE and HCLE cells were followed by live cell time lapse microscopy. Images were taken every 10 minutes until 100 images were acquired (16 hours, 40 minutes). As shown in the schematic, PHLE and HCLE cells were treated with 0.0025% MMC for 3 hours, washed, and re-fed with media lacking MMC and allowed to recover overnight; the following day, live cell imaging was performed as described in the Methods section. Relief contrast microscopic images show the red tracks taken by 10 cells within each field for control and MMC treated cells. Over 30 cells per variable were tracked and migration rates quantified. Data indicate that MMC treated HCLE and PHCE cells migrate significantly slower than control cells. Control and MMC treated HCLE and PHCE cells were, washed, and allowed to recover overnight in media without MMC. Cells were trypsinized and equal numbers of cells allowed to adhere to tissue culture plates coated with a mixture of FN and collagen I for 30 minutes. Significantly fewer MMC treated cells adhere compared to control cells. (**B**) Control and MMC treated HCLE cells were washed and allowed to recover overnight in media without MMC. Cells were then washed 3 times and dilute trypsin (1:15) added. The number of attached cells remaining over time after initiation of trypsinization was determined at 30, 45, 60 and 90 minutes. Fewer cells remain adherent after MMC treatment compared to controls. The numbers of cells remaining attached and their expression of ki67 were determined 24 hr after MMC treatment. Mag bar in A = 12 μm.

Live cell tracking studies were performed to determine the rate of epithelial cell migration after MMC treatment. For these studies, both primary (PHCE) and immortalized (HCLE) corneal epithelial cells were grown to 70–80% confluency before being treated with 0.0025 μg/mL MMC for 3 hours. After treatment, the media containing MMC was removed, cells were washed with PBS, and media added without MMC. Random, non-directional cell movement of treated and untreated cells was assessed every 10 minutes until 100 images were obtained (16 hours, 40 minutes). Treating both HCLE and PHCE cells with 0.0025 μg/mL of MMC for 3 hours significantly reduces cell migration rates following removal of MMC from the media (Fig. 1A).

Changes in cell migration rates can be caused by altered cell substrate adhesion. Equal numbers of control and MMC-treated PHCE and HCLE cells were allowed to adhere for 30 minutes to BSA or to a mixture of fibronectin and collagen I. MMC-treated cells were allowed to recover overnight in media without MMC prior to performing adhesion studies. Figure 1A shows that adhesion of both PHCE and HCLE cells was significantly reduced after MMC treatment compared to control cells. The remaining studies were performed using HCLE cells. PHCE cells require 2x the concentration of BPE (50 μg/mL) and 25x as much EGF (5 ng/mL) for optimal growth compared to HCLE cells. Growing corneal epithelial cells in media with lower levels of growth factors enhanced our ability to identify molecules secreted into the media. For this reason, and since migration and adhesion were reduced by MMC in both PHCE and HCLE cells, we performed the remaining experiments using HCLE cells.

When adherent cells detach from substrates during trypsinization, cell surface proteins including integrins and growth factor receptors become internalized which can induce adhesion dependent cell death (anoikis); cells that are slower to re-express integrins on their surface after being placed in suspension will be less adherent than control cells. To confirm that MMC-treated cells were less adherent, we also performed cell detachment assays (Fig. 1B). Control and MMC-treated HCLE cells were allowed to recover overnight in media lacking MMC before being treated with dilute trypsin to induce detachment. Detached cells were aspirated from wells at 30, 45, 60, and 90 minutes after dilute trypsin was added and adherent cells were stained with crystal violet. Data were normalized relative to wells with cells that had not been treated with trypsin. Interestingly, almost 100% of the control cells remained adherent at 30 and 45 minutes after addition of dilute trypsin but significantly fewer MMC-treated cells remained attached: 65% and 75% at 30 and 45 minutes, respectively. The percentage of adherent cells remaining by 60 and 90 minutes in control samples was 25% and 10% and in MMC-treated cells was 10% and 5%, respectively. Thus, both the adhesion and detachment assays showed that MMC-treated HCLE cells were not as adherent as control cells.

### Significantly fewer total HCLE and ki67 positive cells are present 24 hours after transient MMC treatment

To determine whether transient MMC treatment leads to HCLE cell death and/or altered cell proliferation, we next assessed the number of cells and the number of ki67+ cells present 24 hr after MMC treatment. Results are shown in Fig. 1B. Transient treatment of HCLE cells with MMC results in a significant decrease of approximately 25% in both the number of DAPI+ nuclei and the number of ki67+ cells per field at the 24 hr timepoint. ki67 expression increases in S phase and is maintained in MMC treated cell cycle arrested cells. During cell tracking, we did not observe increased numbers of non-motile or detaching cells after MMC treatment. We conclude that MMC induces 20–25% of cells to detach during or within the first few hours after treatment. Over the next 24 hours, cells continue to remain viable, migrate, and replicate their DNA at levels similar to controls.

### Conditioned media (CM) from MMC-treated HCLE cells (E-CMM) but not control cells (E-CMC) reduces HCLE migration and increases adhesion

**Figure 2:**
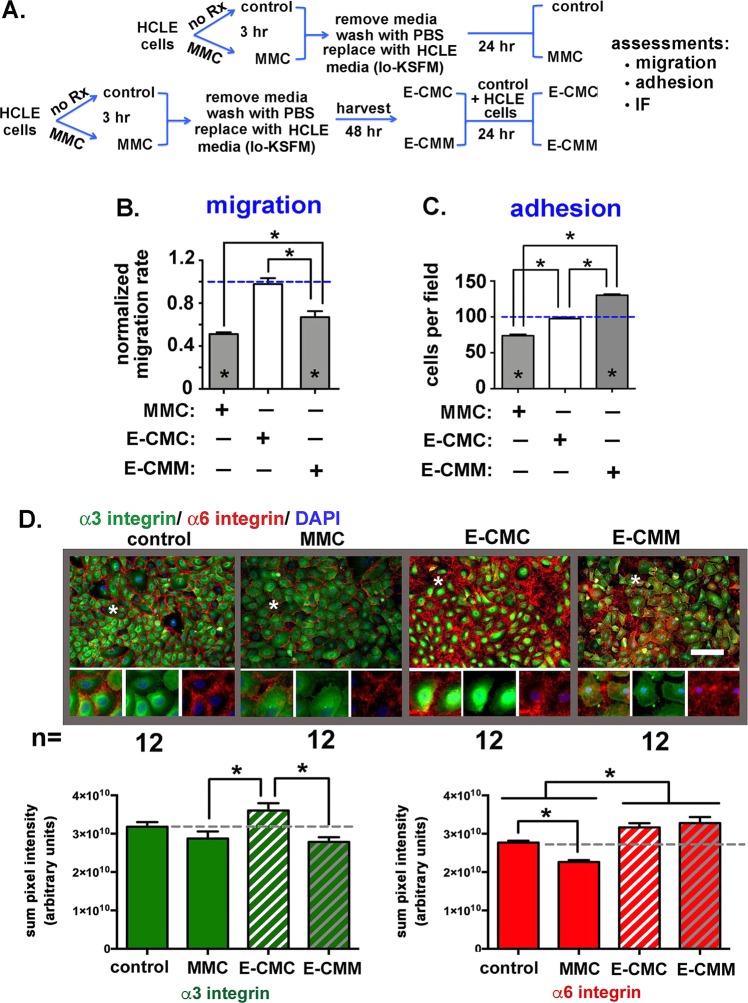
Transient treatment of HCLE cells with MMC for 3 hours induces secretion of molecules into their media that reduce HCLE adhesion, increase migration, and alters expression of α3 and α6 integrins. (**A**) The schematic diagram shows how conditioned media was prepared from control (E-CMC) and MMC treated (E-CMM) HCLE cells and used to assess cell migration and adhesion in HCLE that were not exposed to MMC. (**B**) Random cell migration rates were determined by time lapse microscopy performed as described in Fig. 1. Data are expressed normalized against the migration of untreated control cells incubated in standard HCLE media. Direct MMC-treated cells were included in the analysis. E-CMC did not alter HCLE cell migration rates compared to controls whereas E-CMM significantly reduced the migration rate of HCLE cells. **C**. Cells grown in E-CMC and E-CMM overnight were used in adhesion assays along with controls and cells treated with MMC for 3 hours that were allowed to recover overnight. As expected, direct treatment with MMC reduced adhesion. Cells grown overnight in E-CMC adhered similarly to control cells whereas cells treated with E-CMM have increased adhesion. In (**B,C**) asterisks within bars indicate significant differences compared to untreated control cells whereas asterisks above bars indicate significant difference between treatment groups. (**D**) Indirect immunofluorescence was performed to assess the localization and expression of α3 and α6 integrins in HCLE cells treated with MMC for 3 hours, as well as in HCLE cells incubated in E-CMC and E-CMM. The regions highlighted by asterisks in each panel were magnified 3-fold and presented in the insets to make it easier to appreciate differences in the localization of α3 and α6 integrins. Expression was quantified for each integrin in 12 separate fields in 3 separate wells for control and MMC and 8 separate fields in 3 separate wells for E-CMC and E-CMM and data are expressed as the summation of pixel intensities per field. There is less α3 integrin expressed in HCLE cells after MMC treatment, but the difference is not significant compared to controls. Growing cells in E-CMC increases α3 integrin expression relative to MMC treated cells whereas E-CMM reduces α3 integrin. There is significantly less α6 integrin expressed after MMC treatment. Growing cells in E-CMC and E-CMM increased α6 integrin expression relative to both controls and MMC treated cells. The magnification bar equals 40 μm.

Next, we asked whether treating HCLE cells with MMC caused them to secrete factors (proteins and other molecules) that would impact cell migration and adhesion of cells not directly exposed to MMC. As shown schematically in Fig. 2A, for these experiments, control HCLE cells were grown until subconfluent in HCLE media. Media was then replaced with conditioned media from either control HCLE cells (E-CMC) or with conditioned media from MMC-treated HCLE cells (E-CMM). The cell migration data presented in Fig. 2B includes data from control and transiently MMC treated cells. Cells were tracked every 10 minutes until 100 images were acquired. As shown, HCLE cells grown in E-CMC migrated at the same rate as controls grown in HCLE media (dashed blue line) but cells grown in E-CMM migrated significantly slower compared to controls and cells treated with E-CMC. While the reduction in migration observed in cells grown in E-CMM was significant, it was less than that seen in cells directly treated with MMC for 3 hours and allowed to recover overnight.

Adhesion experiments were next performed on cells grown in control media, after a 3-hour MMC treatment, as well as in cells in E-CMC and E-CMM. Cell adhesion data, normalized against control cells (dashed blue line), are presented in Fig. 2C. Compared to controls, cells treated with E-CMC adhered similarly whereas those treated with E-CMM were more adherent than control cells.

### MMC treatment alters HCLE expression of α3 and α6 integrins and increases the deposition of LN332 in the extracellular matrix

Epithelial cell adhesion and migration are mediated by integrins that attach to ECM proteins synthesized and assembled by the epithelial cells. A key matrix protein mediating adhesion and migration in epidermal and corneal epithelial cells is LN332; *in vitro* epithelial cells secrete LN332 which sticks to coated tissue culture plastic and provides a matrix for the cells to migrate on. The integrins that associate with this laminin are α3β1 and α6β4^22^. α3β1 mediates adhesion to LN332 via the actin cytoskeleton. By contrast, α6β4 mediates adhesion via intermediate filaments forming tight rivet-like adhesions to LN332 referred to as stable anchoring contacts (SACs)^23,24^. When α6β4 at the epithelial cell basal membrane is bound to LN332 in SACs, integrins form dense clusters that prevent antibodies against α6β4 and LN332 from binding. While α3β1 also associates with LN332, it does not cluster within SACs. α3β1 binding to LN332 on epithelial cells modulates epithelial cell migration and hemidesmosomal assembly via a process referred to as transdominant inhibition by competing with α6β4 for binding to LN332^25^.

We next stained control and MMC-, E-CMC-, and E-CMM-treated cells with antibodies against α3 and α6 integrins (Fig. 2D). α3 integrin localized at perinuclear and cytoplasmic locations including the basal region directly beneath DAPI+ nuclei. While less α3 integrin was present in the MMC-treated cells compared to controls, the difference was not significant. E-CMC treated cells had more α3 integrin than cells directly treated with MMC or with E-CMM. Staining cells for α6 integrin revealed no perinuclear staining indicating that intracellular stores of α6 integrin in these cells are low. Most of the α6 integrin stained was present around cells; unlike α3, there was no staining for α6 integrin beneath the DAPI+ cell nuclei suggesting that antibodies against α6 integrin did not penetrate to the basal aspect of the cells. There was significantly less α6 integrin present in MMC-treated cells compared to controls; E-CMC and E-CMM treated cells expressed more α6 integrin than control or MMC-treated cells.

**Figure 3:**
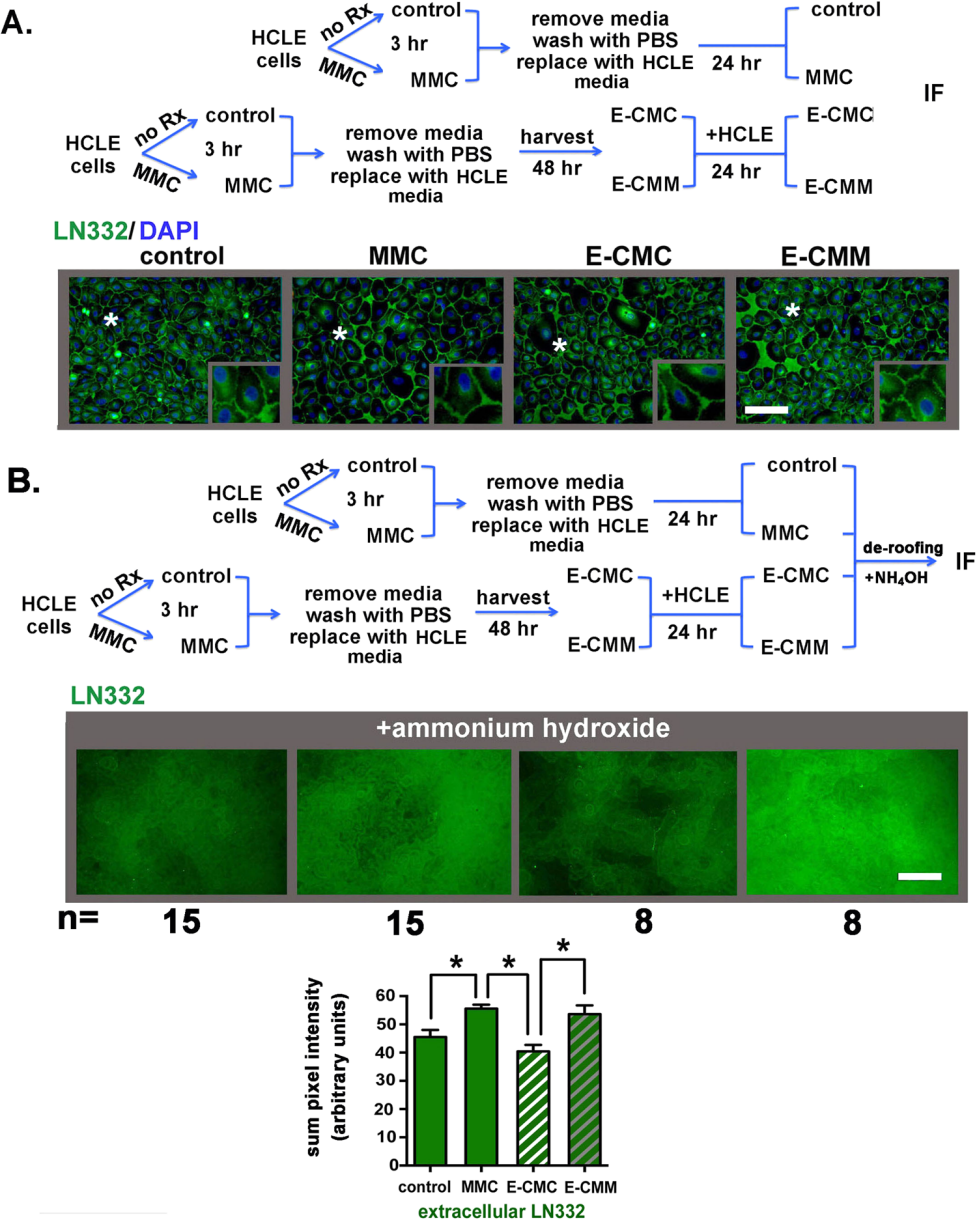
Transient MMC treatment and growing cells in E-CMM increases the extracellular deposition of LN332 by HCLE cells. (**A**) Indirect immunofluorescence was performed to assess the localization and expression of the α3β1 and α6β4 integrin ligand LΝ332 in HCLE cells treated as described in the schematic. LN332 appears excluded from the basal surface of HCLE cells. (**B**) To determine whether the absence of LN332 underneath HCLE cells is due to tight adhesion of cells to LN332 and the inability of the LN332 antibody to reach its epitope, cells grown as described in the schematic were treated with ammonium hydroxide to remove cells, a method referred to as de-roofing, which leaves the ECM behind. Indirect immunofluorescence was performed to assess the deposition of LΝ332 by HCLE cells treated with MMC for 3 hours and in HCLE cells incubated in E-CMC and E-CMM. Deposition of LN332 by HCLE cells was quantified in 15 separate fields in 3 separate wells for control and MMC treated cells and in 8 separate fields in 3 separate wells for E-CMC and E-CMM treated cells. Data are expressed as the summation of pixel intensities per field. Data show that LN332 is excluded from the basal surface of the epithelial basal cells and there is more LN332 deposited by MMC and E-CMM treated cells compared to control and E-CMC treated cells. The magnification bar equals 40 μm.

Staining cells for LΝ332 (Fig. 3A) reveal both perinuclear localization as well as staining around cell borders but little staining beneath the DAPI+ nuclei. The absence of α6 integrin (see Fig. 2D) and LN332 beneath the HCLE cell nuclei suggest that LN332 was present in SACs that prevent antibodies from reaching their epitopes. To determine if this was the case, 0.2 M ammonium hydroxide (NH4OH) was added to replicate plates containing control MMC-, E-CMC-, and E-CMM-treated cells; this treatment, referred to a de-roofing^26,27^, lyses cells and removes cells and debris leaving the ECM behind adhered to the tissue culture surface. ECM preparations were fixed and stained to detect LN332. In Fig. 3B, representative images are shown for each variable assessed; LN332 was quantified in 15 images for control and MMC-treated cells and in 8 images from E-CMC and E-CMM treated cells. Data confirm that LN332 localizes at cells basal aspect and antibodies were excluded from those sites in permeabilized cells. Both direct MMC treatment and growing cells in E-CMM but not E-CMC significantly increased LN332 deposition by HCLE cells.

### MMC-treated HCLE cells upregulate expression of genes associated with senescence and secrete more cytokines into conditioned media compared to control cells

**Figure 4:**
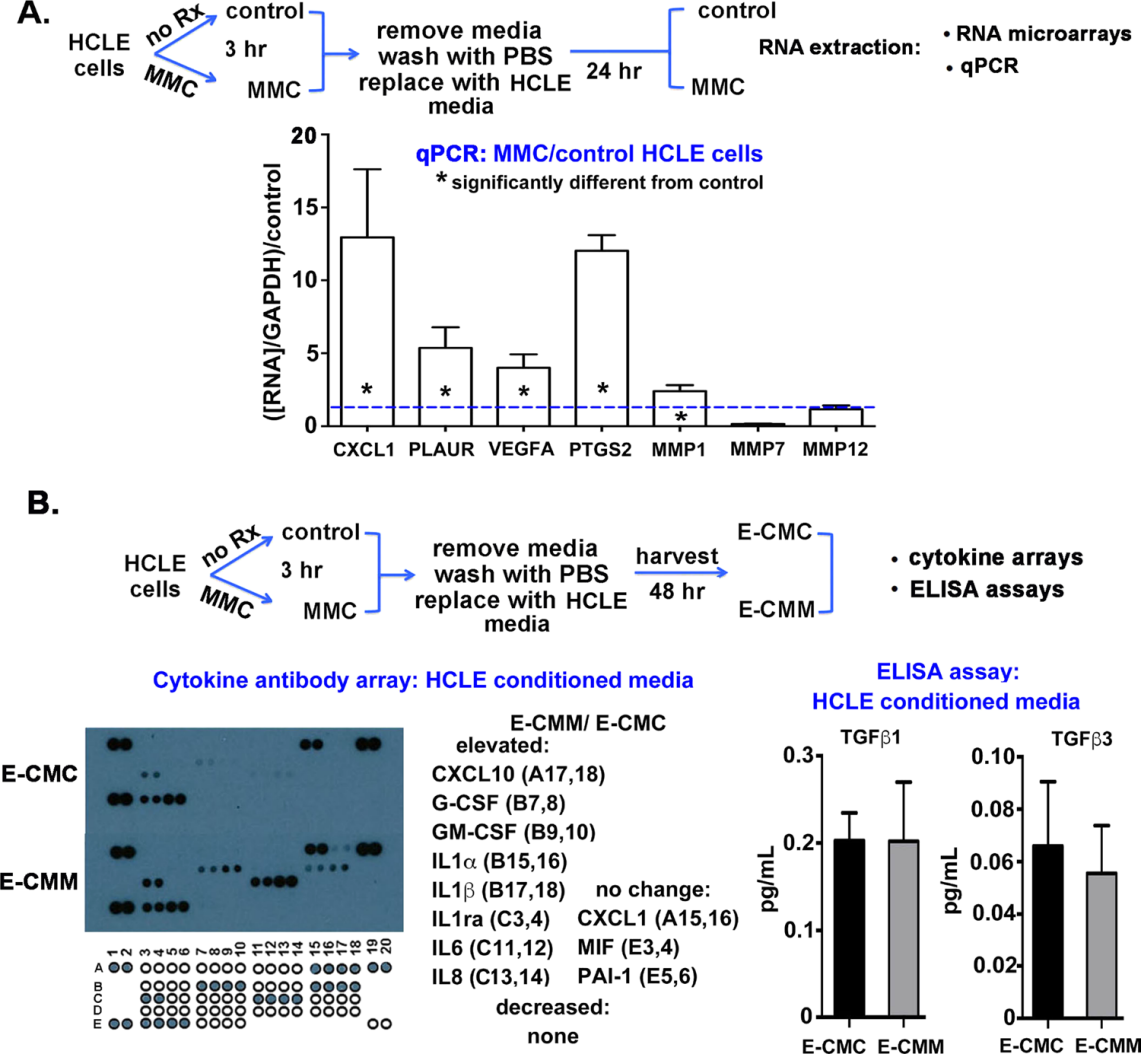
While qPCR and cytokine arrays show that MMC treatment elevates expression of several RNAs and proteins associated with cell migration and senescence, HCLE secretion of TGFβ1 and TGFβ3 are not altered by MMC treatment. (**A**) As indicated by the schematic, RNA was isolated from control and MMC treated cells allowed to recover overnight after a 3-hour MMC treatment. RNAs whose expression was found altered in human wound healing and cell migration RNA arrays were assessed by qPCR. Asterisks within bars indicate significant differences compared to untreated control cells indicated by the blue dashed line. While expression of MMP7 and MMP12 were not altered in MMC treated cells, expression of MMP1, CXCL1, PLAUR, VEGFA, and PTGS2 were elevated significantly with CXCL1 and PTGS2 both elevated more than 10-fold. Data were normalized against expression of the indicated RNA in control cells. (**B**) Conditioned media (CM) from equal numbers of control and MMC-treated HCLE cells was obtained as indicated in the schematic to determine whether secretion of cytokines is altered by MMC treatment. Cytokine arrays were performed twice using CM from cells obtained from 2 independent experiments. While most cytokines were below detectable, 8 were elevated significantly including CXCL10, G-CSF, GM-CSF, IL1α, IL1β, IL1ra, IL6, and IL8 and 3 were shown to not change in their secretion including CXCL1, MIF, and PAI-1. (**C**) TGFβ1 and TGFβ3 levels in E-CMC and E-CMM were quantified using ELISA assays. No significant differences were seen.

The results presented thus far indicate that in response to a transient (3-hour) MMC treatment, corneal epithelial cells migrate slower, are less adherent, and show differences in integrin expression and LN332 deposition. In addition, data obtained using conditioned media from control and MMC-treated cells shows that molecules secreted into E-CMM have the ability to alter cell migration and adhesion of HCLE cells. HCLE cells were next treated with MMC for 3 hours, washed, and allowed to recover in media without MMC overnight; RNA from control and MMC-treated cells was extracted and used in human RNA arrays designed for the study of cell motility and wound healing. While the expression of most mRNAs assessed was not significantly different 24 hours after MMC treatment, several mRNAs including MMP1, MMP7, and MMP12 as well as PLAUR (urokinase receptor), CXCL1, VEGFA, and PTGS2 (prostaglandin-endoperoxide synthase 2 or cycloxygenase-2) were increased in expression after MMC treatment; none were decreased in the arrays. Primers were then used for these mRNAs and qPCR experiments performed to verify whether RNA expression was altered. Data are presented in Fig. 4A for RNA expression for MMC-treated cells after normalization against GAPDH and control cell RNA. Expression of genes for MMP1, CXCL1, PLAUR, VEGFA, and PTGS2 was elevated in MMC-treated cells; no significant changes were seen for MMP7 and MMP12.

**Figure 5:**
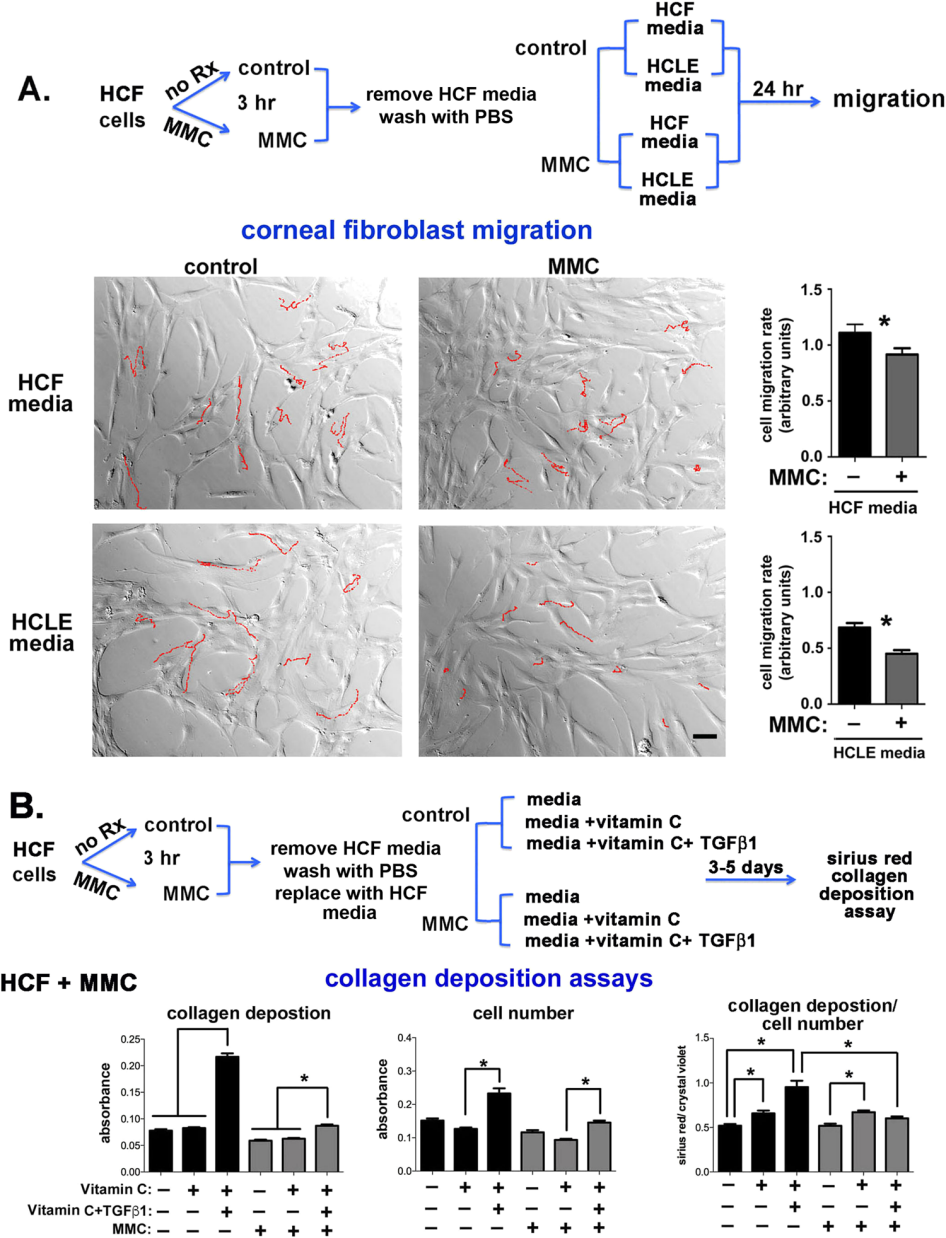
Transient MMC treatment decreases cell migration and TGFβ1 mediated collagen deposition by human corneal fibroblasts (HCFs). (**A**) As indicated in the schematic, fibroblasts were treated with MMC for 3 hours, washed, and re-fed with media lacking MMC and tracked overnight. Two different media types were used for these studies: HCF media containing 10% FCS, and HCLE defined media lacking serum. The following day, live cell imaging was performed. Results show that while HCFs migrated faster in media containing serum, MMC decreased HCF migration rates significantly in both types of media. (**B**) As indicated in the schematic, control and HCFs were treated with MMC for 3 hours, washed, and re-fed with HCF media lacking MMC, media supplemented with vitamin C, and media with both vitamin C and TGFβ1. Results show that while vitamin C and TGFβ1 enhance collagen deposition in control cells, treating cells with MMC prevents TGFβ1 from enhancing collagen deposition by HCFs.

We next used E-CMC and E-CMM on cytokine antibody arrays. Data are presented in Fig. 4B and show that while E-CMC and E-CMM secreted similar amounts of CXCL1, MIF, and serpine 1 (PAI-1), another 8 cytokines assessed —CXCL10, G-CSF, GM-CSF, IL1ra, IL1α, IL1β, IL6, and IL8 —were present in higher amounts in E-CMM compared to E-CMC. The remaining 22 cytokines assessed were undetectable in both E-CMC and E-CMM. Cytokine arrays were performed twice on E-CMC and E-CMM from HCLE cells and once on E-CMC and E-CMM from PHCE cells (data not shown). Results presented in Fig. 5B are representative of those obtained for both HCLE and PHCE cells.

Fibrosis in corneal fibroblasts is known to be regulated by TGFβ signaling. Data indicate that TGFβ1 is pro-fibrotic whereas TGFβ3 expression enhances regeneration without scar formation^28,29^. To determine whether MMC induces HCLE cells to alter their secretion of TGFβ1 and TGFβ3, we next performed ELISA assays using E-CMC and E-CMM (Fig. 4B). While secretion of both molecules by HCLE cells was low, it is quantifiable; we saw no significant differences in E-CMC and E-CMM. ELISA was performed on E-CMC and E-CMM from two independent experiments.

These RNA and cytokine antibody array data together show that MMC treatment induces HCLE cells to up regulate several protease and cytokine mRNAs and to secrete cytokines consistent with a senescence associated secretory phenotype (SASP); the increased secretion persists for at least 48 hours after a transient 3-hour MMC treatment. Differences in cytokine secretion by cells transiently treated with MMC were accompanied by decreased cell migration and reduced adhesion; HCLE cells exposed to E-CMM also migrated slower and they showed increased adhesion thus indicating that MMC exerts direct and indirect effects on corneal epithelial cell migration and adhesion.

### Transient MMC treatment of human corneal fibroblasts (HCFs) reduces their migration and attenuates the ability of vitamin C and TGFβ1 to increase collagen deposition

We next assessed the impact of direct transient MMC treatment (3 hours) on HCF cell migration. HCFs were grown to 70%-80% confluence in standard HCF media supplemented with 10% FBS. HCFs were treated with the same concentration of MMC (0.0025 μg/mL) used previously for PHCE and HCLE cells for 3 hours in HCF media. Cells were then grown in in standard HCF media with 10% serum or in HCLE media and used for time lapse cell migration studies. The rationale for doing experiments using HCLE media was to provide justification for using conditioned media from HCLE cells on HCFs. It was important to document that the HCF cell phenotype after MMC treatment was similar in both medias. While HCF migration rates were slower in HCLE media lacking serum compared to HCF media with 10% serum, MMC treatment reduced migration rates significantly in cells cultured in both HCF and HCLE media lacking serum (Fig. 5A).

HCFs stimulated by vitamin C and TGFβ1 have the ability to assemble and deposit collagen into their surrounding matrix^29,30^. Experiments were performed to assess collagen deposition in control and transiently (3 hours) MMC-treated HCFs grown in either HCF media only, or HCF media supplemented with vitamin C or with both vitamin C and TGFβ1 for 3 days. Collagen deposition was examined in triplicate wells per variable using a Sirius Red assay; Sirius red is a well characterized azo dye that binds to collagen type I and III and is used in histology to assess fibrosis. Data are expressed as collagen deposition per well, cell density per well, and as the ratio of collagen deposition/cell density in Fig. 5B. Data show that control cells treated with vitamin C and TGFβ1 increased their deposition of collagen. MMC-treated cells increased their proliferation rate significantly when exposed to vitamin C and TGFβ1. However, when collagen deposition was normalized by cell number, it was clear that vitamin C and TGFβ1 do not increase collagen deposition in MMC-treated HCFs.

**Figure 6:**
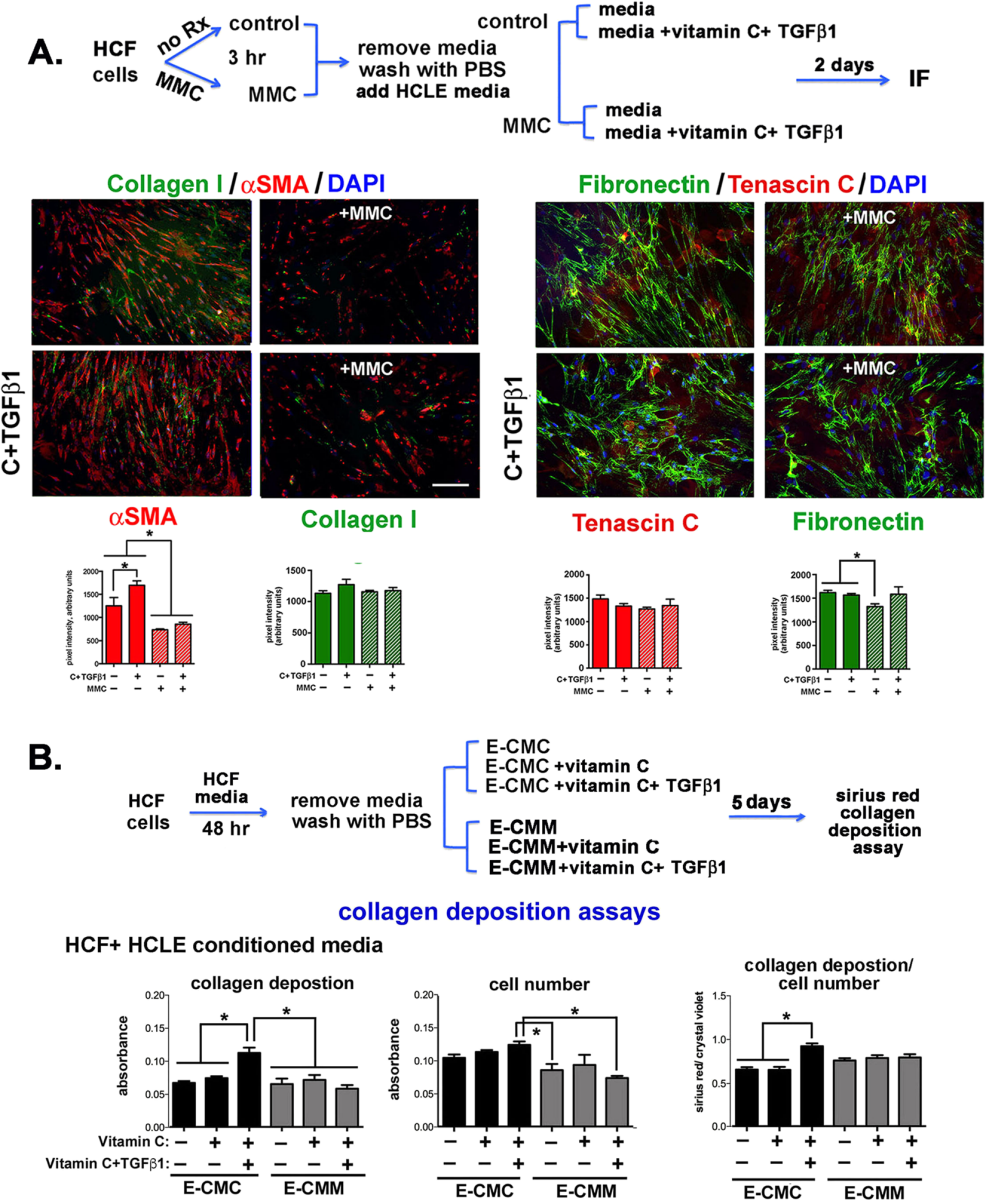
MMC reduces αSMA and fibronectin localization and expression in HCFs and conditioned media from MMC treated HCLE cells blocks TGFβ1-induced collagen deposition. (**A**) As indicated in the schematic, control and MMC-treated HCFs were grown in HCF media and in media supplemented with vitamin C and TGFβ1 for 48 hours. Cells were fixed, permeabilized and stained with antibodies against αSMA (red) and collagen I (green) and TN-C (red) and fibronectin (green). In addition, nuclei were stained with DAPI (blue). Data show that 48 hours after MMC treatment, αSMA expression and FN fibril formation in HCFs is reduced. The magnification bar = 40 μm. (**B**) To determine whether exposure to proteins secreted by MMC-treated HCLE cells impacts collagen deposition by HCFs, E-CMC and E-CMM was added to HCF cultures and collagen deposition assessed. Data show that the addition of vitamin C and TGFβ1 to E-CMM did not induce collagen deposition but addition of vitamin C and TGFβ1 to E-CMC increased collagen deposition by HCFs significantly.

To better understand how MMC blocks vitamin C and TGFβ1 mediated collagen deposition, HCFs were treated with MMC and grown in media with and without vitamin C and TGFβ1 supplementation for 2 days. Cells were then fixed and the expression of collagen I (CN-I) and smooth muscle action (αSMA) as well as fibronectin (FN) and tenascin C (TN-C) assessed by immunofluorescence. Expression of αSMA by corneal fibroblasts is associated with the myofibroblastic phenotype and fibrosis^31–33^, FN fibril formation precedes collagen deposition during fibrosis^34–36^, TN-C modulates ECM assembly^37^, and CN-I, FN, and TN-C accumulate within scar tissues^33^. Data are presented in Fig. 6A. HCFs treated with MMC express significantly less αSMA compared with control cells; supplementing media with vitamin C and TGFβ1 increased expression of αSMA significantly in control but not in MMC treated cells. CN I had yet to accumulate significantly around HCFs and no differences were seen for any of the variables assessed. HCFs treated with MMC expressed less fibronectin compared to control cells with or without vitamin C and TGFβ1 supplementation. TN-C expression remained the same over all experimental conditioned assessed. Thus, MMC treatment reduced αSMA and fibronectin expression by HCFs; MMC also impaired the ability of vitamin C and TGFβ1 to induce αSMA in HCFs.

### Conditioned media from MMC-treated HCLE cells but not from MMC-treated HCFs attenuates vitamin C and TGFβ1 mediated collagen deposition by HCFs

We next asked whether treating HCFs with E-CMM would alter collagen deposition in the matrix by growing HCFs for several days in E-CMC and E-CMM alone and in E-CMC and E-CMM supplemented with vitamin C or with both vitamin C and TGFβ1. Data in Fig. 6B show that HCFs treated with E-CMC supplemented with both vitamin C and TGFβ1 increased their deposition of collagen but HCFs treated with E-CMM supplemented with both vitamin C and TGFβ1 did not.

**Figure 7:**
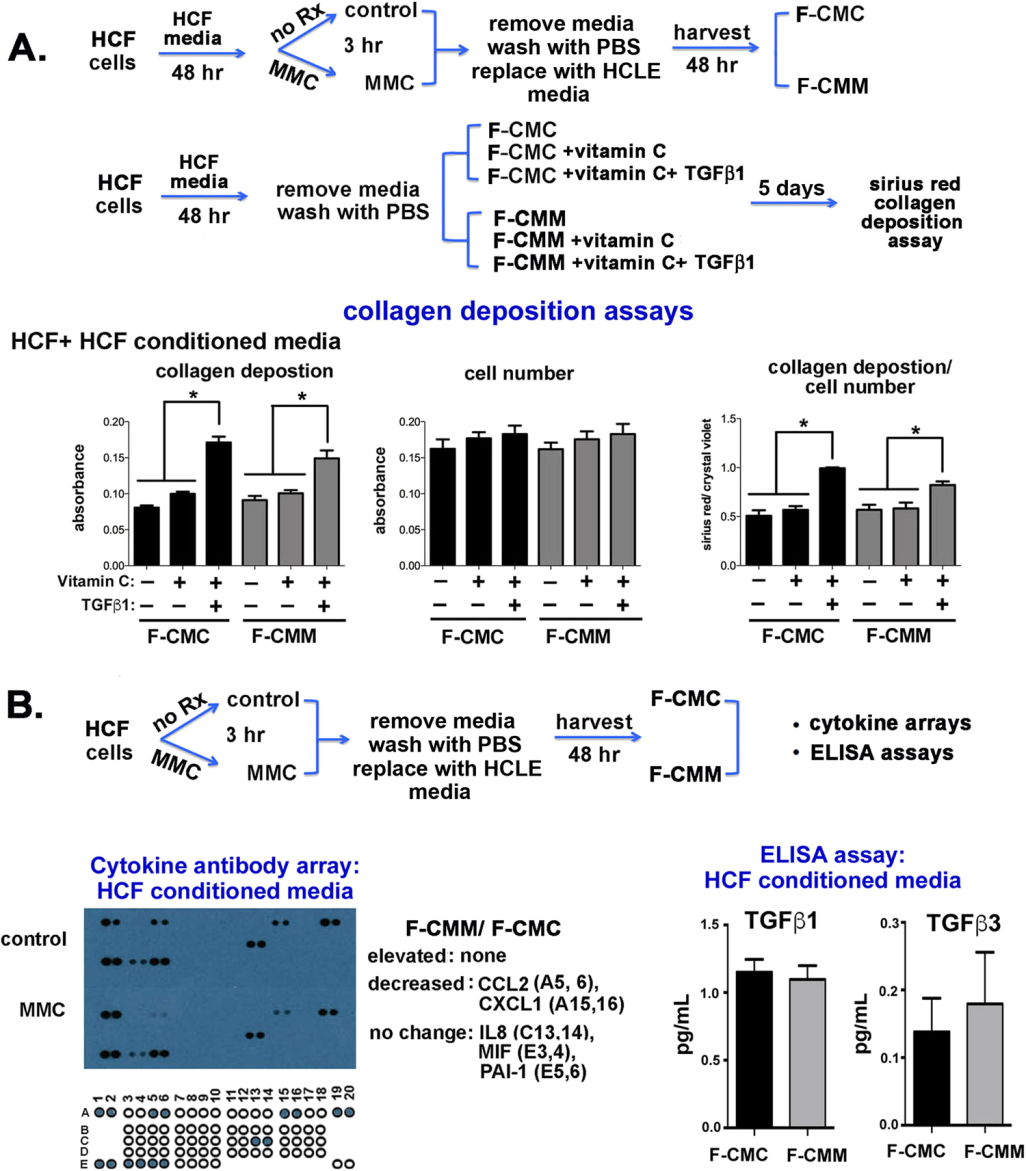
Conditioned media from MMC treated HCFs supports TGFβ1-induced collagen deposition but does not contain elevated levels of cytokines. (**A**) To determine whether exposure to proteins secreted by MMC-treated HCFs impacts collagen deposition by HCFs, F-CMC or F-CMM was added to HCF cultures with and without supplementation with vitamin C and TGFβ1 and collagen deposition assessed as shown schematically. Data show that the addition of vitamin C and TGFβ1 to both F-CMC and F-CMM significantly increases collagen deposition by HCFs. (**B**) Cytokine arrays were used to determine whether HCFs alter their secretion of cytokines into CM after MMC treatment. Data show that similar amounts of IL8, MIF, and PAI-1 are secreted into F-CMC and F-CMM and less CCL2 and CXCL1 is secreted into F-CMM. (**C**) To determine whether HCFs secrete different amounts of TGFβ1 and TGFβ3 into their media after MMC treatment, ELISA assays were performed on F-CMC and F-CMM without supplementation. No differences were observed for either growth factor.

The data presented in Fig. 5B show that direct treatment of HCFs with MMC for 3 hours reduced vitamin C and TGFβ1 induced collagen deposition by HCFs and the data in Fig. 6B show that indirect treatment of HCFs with E-CMM also prevents vitamin C and TGFβ1 induced collagen deposition by HCFs. We next obtained CM from control and MMC treated HCFs and used it for Sirius Red assays with HCFs and HCFs supplemented with vitamin C or both vitamin C and TGFβ1. Data in Fig. 7A show that both F-CMC and F-CMM supplemented with vitamin C and TGFβ1 induced significant increases in collagen deposition by HCFs. While factors (proteins and other molecules) secreted by MMC treated HCLE cells prevent vitamin C and TGFβ1 from enhancing collagen deposition in HCFs, factors secreted by MMC treated HCFs do not.

We next studied whether HCFs treated with MMC also secrete more cytokines into their media using human cytokine arrays. F-CMC and F-CMM were applied to human cytokine antibody arrays designed to detect 32 human cytokines. Data are presented in Fig. 7B and show that while control and MMC-treated HCFs secreted the same amount of IL8, MIF, and serpine 1 (PAI-1), MMC-treated HCFs secreted less CCL2 and CXCL1. None of the cytokines detected were secreted in elevated levels by MMC-treated HCFs. The remaining 27 cytokines assessed were undetectable in CM from control and MMC treated HCFs. To determine whether F-CMC and F-CMM contained different levels of TGFβ1 and TGFβ3, we also performed ELISA assays (Fig. 7B). HCFs secreted 5–6-fold more TGFβ1 and 2-3-fold more TGFβ3 into media than HCLE cells (compare ELISA data in Fig. 7B for HCFs to that in Fig. 4B for HCLE cells). Yet data show that transient treatment with MMC did not alter the level of TGFβ1 or TGFβ3 secreted into CM.

## Discussion

Earlier studies showing that a brief treatment of human fibroblasts with MMC lead to reduced collagen and FN synthesis by human trabecular meshwork fibroblasts formed the basis for the use of MMC in glaucoma filtration and refractive surgery^38^. In our current study, we show that short-term MMC treatment increased LN332 deposition by HCLE cells and attenuated the ability of vitamin C and TGFβ1 to enhance collagen deposition by HCFs. Also, epithelial-derived CMM blocked vitamin C and TGFβ1 mediated collagen deposition in HCFs, but fibroblast-derived CMM did not. These results indicate for the first time that the ability of MMC to reduce fibrosis is due to its ability to both enhance epithelial basement membrane protein deposition by corneal epithelial cells and to reduce expression of αSMA and FN by corneal fibroblasts.

The long-term impact of MMC use on corneal epithelial stem cells and corneal endothelial cells has given pause to its wide spread use in the clinic. Yet, recent reviews continue to document its ability to improve surgical outcomes after refractive surgery^39–41^. Patients experiencing a retinal detachment can develop proliferative vitreoretinopathy (PVR) in which RPE and glial cells proliferate and secrete fibrotic material which exerts traction on the retina and can lead to further vision loss. Treatments for PVR met with limited success. Yet, *in vitro* studies by Kang and colleagues^42^ show that MMC treatment of RPE cells can lead to cell cycle arrest and their apoptosis. *In vivo* studies^43,44^ using MMC to treat PVR have begun to appear.

The ability of MMC to reduce scar formation *in vivo* has long been assumed to be mediated by its ability to attenuate TGFβ signaling in fibroblasts^33,45,46^. Here we show that MMC appears to suppress fibrosis by reducing collagen deposition by corneal fibroblasts and by enhancing deposition of epithelial basement membrane proteins by corneal epithelial cells as shown by the increase in LN332 deposition observed when HLCE cells were induced by direct and indirect treatment with MMC. This knowledge contributes to our understanding of why failure of the epithelial basement membrane to reassemble after refractive surgery and other corneal injuries and pathologies can lead to fibrosis^47–49^. Also, studies by Saikia and colleagues^50^ show that IL1β increases expression of the epithelial basement membrane proteins perlecan and nidogen by corneal stromal cells. Here we find increased secretion of IL1β into media after MMC treatment of HCLE cells but not HCFs. Additional studies will be needed to determine whether exposing HCFs to epithelial cell CMM increases LN332, perlecan, or nidogen expression. The fact that we see no changes in secretion of TGFβ1 or TGFβ3 by MMC-treated HCLE cells or HCFs suggests that other signaling networks in addition to those mediated by TGFβ play roles in the ability of MMC to reduce fibrosis. However, MMC can reduce, directly and indirectly, the ability of TGFβ1 to induce collagen deposition in corneal fibroblasts.

We used the HCLE cell line for the cytokine and matrix deposition studies presented here^51^. Primary corneal epithelial cells are more expensive to grow and undergo replicative senescence if serially passaged. Primary cells also require media containing 25x more EGF and 2x more BPE supplemented in their media compared to HCLE cells. Using HCLE cells allowed us to minimize the growth factors present in the media and maximize differences after MMC treatment. hTERT immortalized human corneal epithelial cells, like numerous other hTERT immortalized cells^52^, retain many of the properties seen in primary cells including their ability to differentiate and secrete cell type specific proteins in ways similar to those seen in primary cells^53,54^. hTERT immortalized fibroblasts and cancer cells respond to genotoxic agents like MMC, elevated ROS, and ionizing radiation by upregulating genes associated with senescence^55,56^; while more resistant than non-immortalized cells, hTERT immortalized cells die after genotoxic stress.

The fact that cancer cells show high levels of telomerase activity makes studying hTERT epithelial cells critical to development of better treatments for epithelial derived cancers. Whether HCLE cells undergo drug induced senescence in response to transient MMC treatment was not known prior to our studies but we show here that HCLE cells exposed transiently to low concentrations of MMC reduce their migration and secrete SASP factors into their media in ways similar to PHCE cells.

To study the impact of direct treatment of epithelial cells and fibroblasts with MMC, we obtained media secreted by cells treated with MMC for 3 hours; cells were allowed to recover after removal of MMC for 48 hours during which time they secreted proteins into the media. By comparing control and MMC-treated conditioned media (CM) from HCLE cells and HCFs here we show that MMC treatment induced epithelial cells to secrete more of several cytokines involved in wound healing into media whereas it reduced cytokine secretion by HCFs. CM from MMC-treated HCLE cells suppressed collagen deposition but CM from MMC-treated HCFs did not. These data support the involvement of corneal epithelial cell cytokine secretion in mediating the ability of MMC to suppress scarring *in vivo*. Our results also implicate secretion of specific corneal epithelial cell cytokines in mediating collagen deposition; additional studies will be required to confirm this.

**Figure 8:**
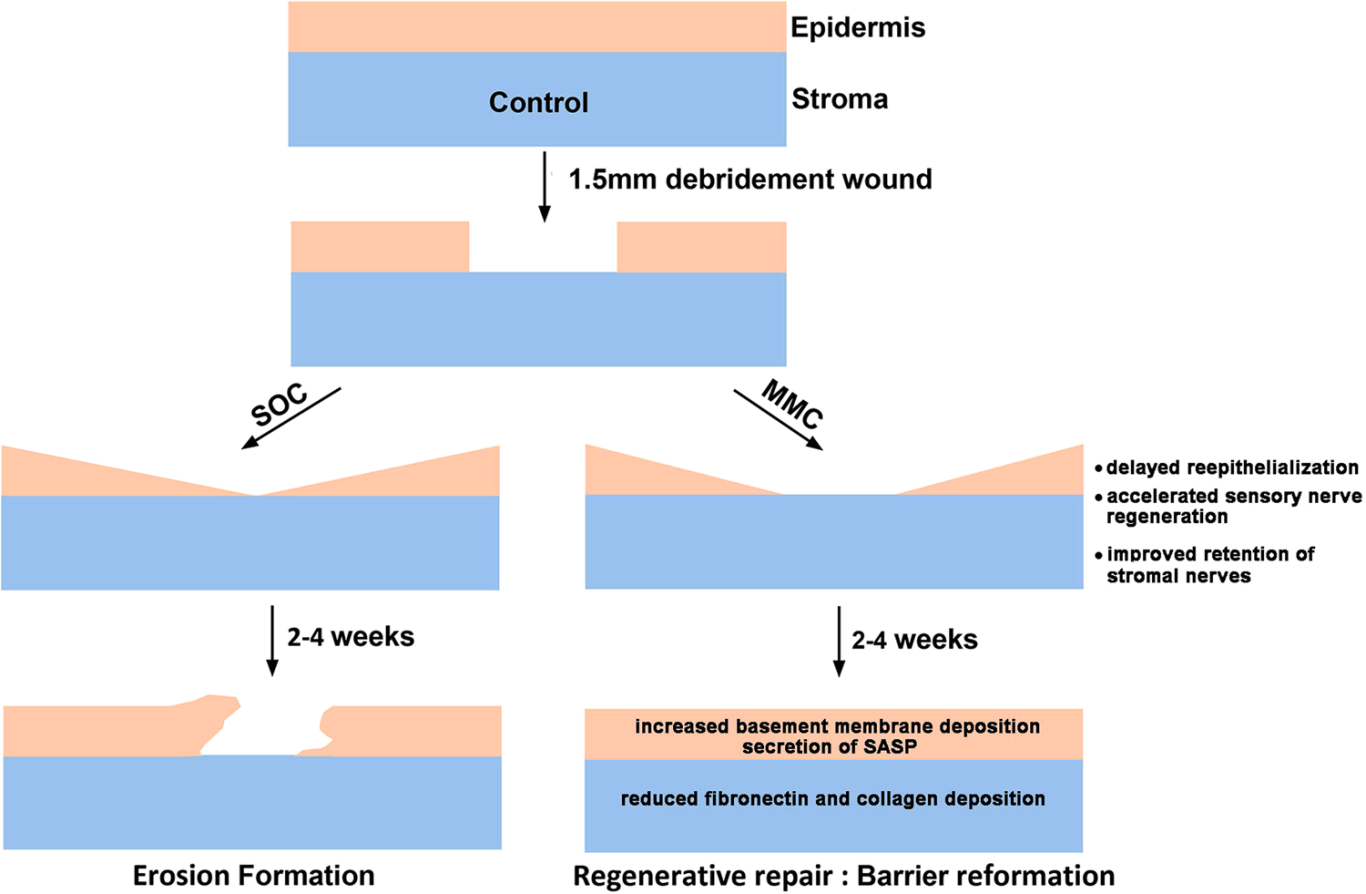
Data from *in vivo* and *in vitro* studies using MMC indicate that corneal epithelial cells play key roles in regulating regenerative wound repair in the cornea. *In vivo* studies have shown that a single prophylactic treatment of MMC enhances regenerative wound repair and RNAseq studies show that epithelial cells upregulate mRNAs for SASP proteins^19,20^. Our *in vitro* studies show that corneal epithelial cells also respond to MMC by upregulating SASP proteins and these proteins are capable of suppressing TGFβ1 induced matrix deposition by corneal fibroblasts. Taken together, these data indicate that corneal epithelial cells as well as corneal fibroblasts play important roles in regulating regenerative, non-scarring, repair of the cornea after injury. SOC = standard of care. SASP = senescence associated secretory proteins.

These *in vitro* data add significantly to *in vivo* data obtained by treating debridement wounded mice with a single does of MMC at the time of injury as shown schematically in Fig. 8. In response to injury, activated epithelial and stromal cells proliferate and migrate into the injured site. Some of these cells were not present when the drug was applied at the time of injury having migrated into the wound area from the periphery; other cells were exposed to MMC directly, became senescent, and ceased proliferating. MMC has a half-life of less than 20 minutes *in vivo* and is degraded at low pH within lysosomes^3^ and yet the long-term impact of MMC on the cornea after refractive surgery persists for several weeks after injury^57^. The *in vitro* data presented here, combined with that in the literature, indicate that the ability of MMC treatment to decrease scar formation and enhance corneal sensory reinnervation is mediated indirectly in the days and weeks following surgery through the secretion of proteins by subpopulations of epithelial and stromal cells that were exposed directly to the drug at the time of injury. These factors are able to increase basement membrane formation by the corneal epithelial cells and reduce fibrosis by corneal fibroblasts by attenuating TGFβ1 signaling. Additional transcriptomics and proteomics studies are needed to fully understand how this drug works.

## Materials and Methods

### Cell culture

Telomerase immortalized human (hTERT) corneal limbal epithelial (HCLE) cells were generated^51^ and validated annually by STR analysis and for the presence of mycoplasma. Stock vials of HCLE cells were thawed at 37 °C and suspended in neutralization medium [500 mL of DMEM/F12 (Gibco #11039-021), 55 ml calf serum (Gibco #A3382001), and 5.5 mL 100X Pen-Strep (Gibco #15140-122)]. Cells were then resuspended in HCLE media which consists of supplemented GIBCO Keratinocyte SFM (Gibco #10724-011); the concentration of BPE used was 25 μg/mL (Gibco 13028-014) and of EGF was 0.2 ng/mL (Gibco 10450-013) with 5 mL 100x Pen-Strep solution). Cells were grown at 37 °C with 7.0% CO_2_ and fed the next day and then every other day until used for experiments. Cells were not maintained in continuous culture; they were expanded as needed and frozen

Primary human corneal epithelial (PHCE) cells were derived as previously described^58^. For the experiments described, cells were thawed at 37 °C and were transferred to neutralization medium (see above). After centrifugation, cells were resuspended in PHCE media consisting of supplemented Gibco Keratinocyte SFM; the concentration of BPE used was 50 μg/ml (Gibco 13028-014) and of EGF was 5 ng/mL (Gibco 10450-013) with 5 mL 100x Pen-Strep solution. Cells were in second passage and grown at 37 °C with 7.0% CO^2^ on tissue culture dishes coated with FNCNI (10 mg/mL FN and 3 μg/mL PureCol Type I CN) as described previously^59^.

Human corneal fibroblasts (HCFs) were obtained as described previously and used for experiments within 3-5 passages^60^. Unless otherwise indicated, HCFs are cultured in Gibco DMEM (#11995-065) media supplemented with 50 mL FBS (Gibco (#A3382001), 5 mL Pen-Strep, 5 mL L-glutamine (#25030-149), and 5 mL non-essential amino acids (#11140-050) per 500 mL bottle. Cells were grown at 37 °C with 7.0% CO_2_.

Conditioned media (CM) was prepared from HCLE cells and HCFs. HCLE cells were plated out in HCLE media in 100 mm^2^ dishes. Fresh media was added after 24 hours; at 48 hours after plating, subconfluent cells were either left alone (controls) or treated with MMC (0.0025%) for 3 hours at 37 °C followed by 2 washes with 1X PBS. Fresh HCLE media without MMC was then added to both control and MMC-treated cells. This CM was harvested 48 hour later, and debris was removed by centrifugation at 3500 rpm for 4 minutes. The number of cells that generated the CM was determined and used to normalize the volume of the CM for the number of cells that generated it. CM was frozen at −80 °C. CM from control corneal epithelial cells is referred to as E-CMC and CM from MMC-treated epithelial cells as E-CMM. To prepare CM from HCFs, HCFs cells were grown in HCF media in 100 cm dishes until subconfluent. HCFs were then left untreated (controls) or treated with MMC (0.0025%) for 3 hours, washed, and HCLE media was added. Cells were grown for 48 hours, CM obtained, debris removed by centrifugation at 1000 rpm for 5 minutes, CM normalized, and aliquots frozen at −80 °C. CM from HCFs is designated as F-CMC (control HCF CM) and F-CMM (MMC-treated HCF CM).

CM from both epithelial cells and fibroblasts was used for human cytokine antibody arrays (R & D Systems, #ARY005) as per the manufacturer’s instructions. In addition, epithelial and fibroblast cell CM was used with the human TGFβ1 PicoKine ELISA (Bosterbio #EK0513) and human TGFβ3 PicoKine ELISA (Bosterbio #EK1 103) kits.

### Immunofluorescence

Immunofluorescence was performed on HCLEs and HCFs after fixation in 4% paraformaldehyde (PFA) and permeabilization using Triton-x 100 as described previously^61^. For de-roofing experiments to remove cells and leave matrix behind^26^, 0.02 M NH_4_OH prepared fresh in 0.1% Triton X100 was added to cells for 10 minutes at room temperature. Cells were washed in 0.1% Triton X100 to neutralize the matrix; preparations were then fixed in PFA as described above and used for immunofluorescence staining for lamimin-332 as described below. The following antibodies were used: α3 integrin (1:200)^62^, α6 integrin (1:200; GoH3, #sc19622, Santa Cruz Biotechnology), laminin-332 (1:500; LN332; J18; Jonathan Jones, Washington State University), α−smooth muscle actin (1:250; αSMA; # T2547, Sigma), fibronectin (1:500; FN; #R5836, Dr. K.M. Yamada, NIH), collagen type I (1:200; CNI; #234167, Sigma), and tenascin-C (1:250; TN-C; #T3413, Sigma). Species-specific Alexa-fluor secondary antibodies (488, 594, and 647; Jackson Immunosciences) were used at 1:500 dilution in blocking buffer. (see above). Images were acquired using Nikon Eclipse TS2R and quantified using NIS Elements BR v5.00.

### Time-lapse cell migration

Corneal epithelial cells and fibroblasts were seeded onto 24 well plates and allowed to grow for 2 days. Following 3 hr MMC treatment, cells were washed, media without MMC was added, and cells were placed in a temperature and CO_2_ controlled chamber (Tokai Hit, Japan) on an Olympus IX81 research microscope (Olympus America, Melville, NY 11747) equipped with a Proscan motorized stage (Prior Scientific Instruments Ltd., Rockland, MA 02370). Using relief-contrast optics, 10 × images were taken per well every 10 minutes for 16 hour and 40 minutes (100 images). For each variable, triplicate wells were tracked. Images were transferred to a workstation equipped with Metamorph image analysis software (Molecular Devices Corporations, Chicago, IL) where velocities of 20 cells were calculated using the track cell module. A visual basic program was written to assist in data analysis. From each cell tracked, an average velocity, net displacement and total displacement were determined. The processive index (net displacement/ total displacement) was also calculated. To verify that there was no change in velocity over time for each experiment, we also routinely assessed velocity over time for each cell tracked. The Visual Basic script used for these determinations is available from the authors upon request.

### Cell adhesion and detachment assays

HCLE cells were grown in 24-well plates to 70-80% confluency. After 48 hours, cells were treated with 0.0025% MMC for 3 hours and allowed to recover overnight in media without MMC followed by trypsinization. Trypsin was neutralized, and cell numbers were obtained using the coulter counter. Equal numbers of control and MMC-treated cells were plated out in 24-well plates coated with BSA alone or with FNCNI; non-adherent cells were aspirated 30 minutes after incubation at 37 °C. Wells were washed 2x with PBS and stained with crystal violet. The dye was either extracted with 10% acetic acid and the ODs measured in a 96-well plate using the Tecan Infinite series plate reader and Magellan (Tecan) software or the number of cells per well were counted. Data are expressed as adhesion of cells to FNCNI/BSA.

For the HCLE detachment assay, HCLE cells were grown in 6-well plates to 70-80% confluency. After 48 hours, cells were treated with 0.0025% MMC for 3 hours and allowed to recover overnight in media without MMC followed by trypsinization with a 1:15 dilution of 0.25% Trypsin-EDTA. Trypsin was neutralized at 0, 30, 45, 60, and 90 minutes. At each time point, media was aspirated, and 0.5% crystal violet was added to the wells. After removing the excess dye by washing, plates were allowed to air-dry overnight and the crystal violet was extracted with 10% acetic acid. OD was measured in a 96-well plate using the Tecan Infinite series plate reader and Magellan software.

### RNA isolation, qPCR and RNA microarrays

HCLE cells were treated with 0.0025% MMC for 3 hours as described above. 24 hr post treatment, RNA was extracted from control and MMC-treated cells as described previously^63^ using the Arcturus PicoPure RNA Isolation Kit (Applied Biosystems- Thermo Fisher, #12204-01) and then cDNA was synthesized from the mRNA using the iScript cDNA Synthesis Kit (Biorad, #170-8891) and cDNA was used with predesigned microarray plates that assessed genes involved in cell motility and wound healing (Biorad, #10034601 and 10034463) as well as in q-PCR using iTaq Universal SYBR Green Supermix (Biorad, #172-5124) and a CFX384 thermocycler from Biorad. Results then were analyzed using the CFX Manager Software. The following qPCR Primers were used: CXCL1 #qHsaCID0010973, PLAUR # qHsaCID0017227, VEGFA # qHsaCED0043454, PTGS2 # qHsaCID0020933, MMP1 # qHsaCID0017039, MMP7 # qHsaCID0011537, MMP12 # qHsaCED0048099. qPCR results for each RNA assessed were normalized to GAPDH with the deltaCt method and all assays were repeated in triplicate.

### Collagen deposition assay

Deposition of collagen by HCFs was assessed using Sirius Red assay as described^21^ with and without addition of vitamin C (0.5 mM) and TGFβ1 (2 ng/mL) to media. After assessing the amount of Sirius Red dye bound by cells, cell density was assessed using crystal violet. Sirius red was used at a concentration of 0.1% in picric acid and crystal violet was used at 0.1% in 50% ethanol. Data are presented as collagen deposition (Sirius Red dye bound) and cell density (crystal violet bound) as well as after normalization by cell density.
